# Exploring the Association of Smoking and Alcohol Consumption with Presence of and Severe Coronary Artery Calcification

**DOI:** 10.31083/j.rcm2510376

**Published:** 2024-10-23

**Authors:** Yinze Ji, Xiaorong Han, Yingzhen Gu, Jinxing Liu, Yifan Li, Wei Zhang, Aimin Dang, Naqiang Lv

**Affiliations:** ^1^Premium Care Center, Department of Cardiology, Fuwai Hospital, Chinese Academy of Medical Sciences & Peking Union Medical College, National Clinical Research Center for Cardiovascular Diseases, National Center for Cardiovascular Diseases, 100037 Beijing, China

**Keywords:** smoking, alcohol consumption, coronary artery calcification, coronary artery calcification score, interaction

## Abstract

**Background:**

Despite the majority of studies have identified smoking as a risk factor for coronary artery calcification (CAC), some studies have not identified this relationship. Differences on results reached by studies on the association of alcohol consumption with CAC exist. Moreover, studies have almost exclusively investigated the association between smoking and alcohol consumption independently. Whether an interaction effect of alcohol on the association of smoking and CAC exists has hardly been investigated.

**Methods:**

The data of 2431 adult patients who visited Fuwai Hospital, Chinese Academy of Medical Sciences from September, 2001 to December, 2023 and had Agaston coronary artery calcification score (CACS) reported were utilized. Patients who (1) underwent percutaneous coronary intervention, coronary bypass graft and heart transplantation, or (2) were complicated by acute medical conditions, chronic kidney disease or malignant neoplasms were excluded. Data from 1528 patients were eventually analyzed. Logistic regression was employed to investigate the association of smoking and alcohol consumption with presence of CAC and severe CAC. Interaction effects of alcohol consumption history on the association of current smoking and both presence of and severe CAC were examined.

**Results:**

Smoking history was significantly associated with presence of CAC and severe CAC. Current alcohol consumption was also significantly associated with presence of CAC and severe CAC. After adjusting for confounders, alcohol consumption history demonstrated an interaction effect on the association of current smoking with both presence of and severe CAC. Using non-alcohol consumers not smoking at the time of the study as reference, current smokers with an alcohol consumption history suffered from an increased risk of presence of CAC and severe CAC.

**Conclusions:**

Both smoking history and current alcohol consumption were associated with presence of and severe CAC. Alcohol consumption history demonstrated an interaction effect on the association of current smoking with both presence of and severe CAC.

## 1. Introduction

Coronary artery calcification (CAC) has been proven to be associated with increased cardiovascular risk. Current studies have demonstrated that the coronary artery calcification score (CACS) plays an important role in the diagnosis of early, subclinical coronary heart disease [1] as well as risk stratification of diabetic [2], hypertensive [3], elderly [4] individuals and smokers [5]. CAC is associated with stent under-expansion, which in turn is associated with in-stent restenosis and thrombosis [6]. Moreover, CAC is associated with the prognosis of certain non-cardiovascular diseases (e.g., malignant neoplasms, hip fracture and chronic obstructive pulmonary diseases) [7].

Significant heterogeneities exist between individuals without CAC (defined as CACS = 0) and those with CAC (defined as CACS >0). In the Rotterdam Elderly Study [8], every group of patients with CACS >0 was found to be associated with a significant increased risk of adverse cardiovascular events as compared with those with CACS = 0. Statins were found to be associated with a reduction of risk for major adverse cardiovascular events in patients with CACS >0 whereas such an association was not observed in those with CACS = 0 [9]. More importantly, McClelland *et al*. [10] found that at a given time point, after adjusting for confounders, liquor consumption was significantly associated with the severity of CAC in populations with CACS >0; yet no association was found between liquor consumption and CACS >0 in the entire population that encompassed both individuals with (defined as CACS >0) and without (defined as CACS = 0) CAC. This indicated that at a given time point, risk factors associated with the presence of CAC in the entire population and those associated with the severity of CAC in the population with CAC may not necessarily be the same, providing theoretical plausibility of investigating the risk factors for the presence of and severe CAC separately.

Studies to date have generally demonstrated the presence of an association between smoking and CAC, despite the absence of evidence of such associations in certain populations. van der Toorn *et al*. [11] found that smoking was associated with CAC volume increment in female populations while such an association was not found in male populations. Capisizu *et al*. [12] found that smoking was significantly associated with severe CAC. Bagyura *et al*. [13] found that smoking was significantly associated with CACS >100 after adjusting for other risk factors. Kiss *et al*. [14] found that smoking was an independent risk factor of CACS ≥300. Molina *et al*. [15] found that current or former smokers were associated with the presence of CAC as well as higher CACS. Min *et al*. [16] found smoking to be a risk factor in the transformation into CACS >0 at follow-up in individuals with baseline CACS = 0.

Yet, there are also studies that failed to find an association between smoking and CAC in certain respects. The aforementioned study by Kiss *et al*. [14] also found no association between smoking and CACS >0 after adjusting for confounders. A multi-center study conducted by Zhang *et al*. [17] found that smoking history (i.e., former or current smoking) exhibited no significant association in patients receiving hemodialysis or peritoneal dialysis. The aforementioned study by Min *et al*. [16] also found no association between smoking and CAC progression in individuals with baseline CACS >0. Yang *et al*. [18] also found no association between smoking and conversion from CACS = 0 to CACS >0 at follow-up.

Despite the presence of studies investigating the association between alcohol consumption and CAC, their results have been inconsistent, with some studies revealing the association between alcohol consumption and CAC in some respects (e.g., the severity of CAC in CACS >0). McClelland *et al*. [10] found that liquor consumption amount correlated significantly with CACS >0 after adjusting for other risk factors; consumption of two drinks of liquor per day was significantly associated with the severity of CAC in individuals with CACS >0 as well as rapid change (>30 units per year) in CAC during follow-up among individuals with baseline CACS >0; total daily alcohol consumption volume correlated positively with annual change in CACS. The Coronary Artery Risk Development in Young Adults (CARDIA) study revealed that liquor consumption was significantly associated with presence of CAC; heavy drinking (≥14 drinks per week) was an independent risk factor of presence of CAC; the statistically significant increasing trend of likelihood of the presence of CAC as the amount of alcohol consumption increased [19]. Serum calciprotein particle maturation time (T50) is negatively associated with calcification propensity. Eelderink *et al*. [20] found that serum T50 was negatively associated with alcohol consumption, suggesting the positive association between the latter and calcification propensity.

There are also studies that failed to find an association between alcohol consumption and the presence of CAC. The aforementioned study by McClelland *et al*. [10] also found no association between alcohol consumption and baseline presence of CAC. This phenomenon was independent of the type of alcohol. In addition, no J-shaped association was observed between alcohol consumption and baseline presence of CAC. Another study found no association between CAC amount and alcohol consumption in asymptomatic individuals at high risk of coronary heart disease [21]. Alcohol consumption as well as the type and amount of alcohol consumed were all found to be uncorrelated with the presence of CAC in a cohort of healthy American military personnels [22]. A U-shaped association between alcohol consumption amount and risk of severe CAC has also been found [23].

As has been mentioned above, different types of drinks containing alcohol demonstrated different associations with CAC. What complicates the association of alcohol consumption and CAC was that substances coexistent in certain drinks containing alcohol may also exert influence upon the calcification process. An animal study conducted by Liou *et al*. [24] found that xanthohumol, a chemical found in hops, could alleviate vascular calcification mediated by vitamin D and nicotine. Hops, on the other hand, is an indispensable ingredient in producing beer. Therefore, beer consumption could influence the association between smoking and vascular calcification. In a nutshell, the association between alcohol consumption and vascular calcification is complex and may influence the association of other risk factors and vascular calcification. It is therefore necessary to examine the association of current smoking with the presence and severity of CAC separately in patients with and without alcohol consumption history. If there is a difference in risk of an event (e.g., presence of CAC) associated with current smoking between those with and those without alcohol consumption history, then alcohol consumption history can be said to have an interaction effect on the association of current smoking and that event.

In summary, the association between smoking and alcohol consumption with both the presence and severity of CAC has not been fully elucidated. Further investigation into the association of smoking and alcohol consumption and both the presence as well as the severity of CAC is warranted. In particular, examination into the interaction effect of alcohol consumption on the association of current smoking and presence of as well as the severe CAC is warranted.

## 2. Materials and Methods

### 2.1 Study Population

**Fig. 1.**
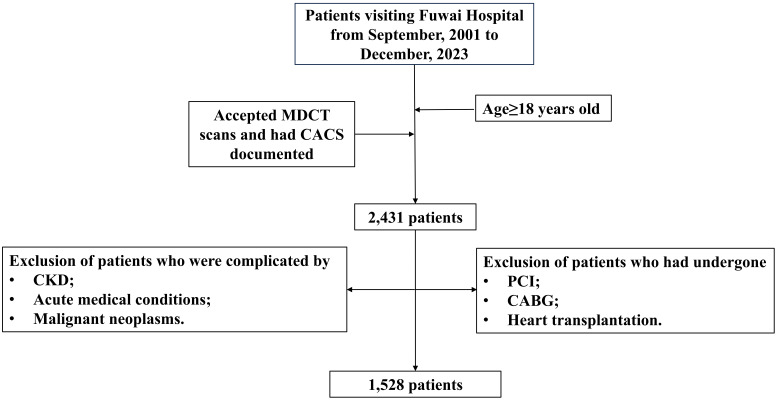
**Schematic representation of the selection of study population**. Abbreviations: CABG, coronary artery bypass graft; CACS, coronary artery calcification score; CKD, chronic kidney disease; MDCT, multidetector row helical computed tomography; PCI, percutaneous coronary intervention.

This study screened 2431 patients that visited Fuwai Hospital, Chinese Academy of Medical Sciences from September, 2001 to December, 2023. Inclusion criteria of this study were as follows: (1) Age ≥18 years old; (2) underwent multidetector row helical computed tomography (MDCT) and had Agaston CACS reported. Exclusion criteria were having undergone percutaneous coronary intervention (PCI), coronary artery bypass graft (CABG), or heart transplantation, complicated by acute medical conditions or chronic kidney disease (CKD) or malignant neoplasms. 1528 patients were eventually selected in this study. The selection process is diagrammatically outlined in Fig. 1.

### 2.2 Data Collection

This study collected patients’ data from the electronic medical record system. Demographic data, history of comorbidities, alcohol consumption and smoking history, medication history, laboratory test results, total Agaston CACS calculated from MDCT were collected. Of note, smoking history was defined as ever (former or current) smoking, current smoking was defined as smoking in the month before the study. Likewise, alcohol consumption history was defined as ever (former or current) alcohol drinking while current alcohol consumption was defined as alcohol drinking in the month before the study. Presence of CAC was defined as total CACS >0 while severe CAC was originally defined as total CACS ≥1000 and then adjusted to be defined as total CACS ≥1100.

### 2.3 Statistical Analyses

Patients were divided into two groups based on the presence of CAC–i.e., patients with CACS = 0 and CACS >0. For continuous variables, normalities were assessed comprehensively by test results from the Shapiro-Wilk test or Kolmogorov-Smirnov test (chosen as appropriate), histogram, probability-probability (P-P) plots and quantile-quantile (Q-Q) plots. Continuous variables that followed normal distributions were reported as mean ± standard deviation and were tested for inter-group difference via *t *tests whereas those that did not follow normal distributions were reported as median (1st quartile, 3rd quartile) and were tested for inter-group difference via Wilcoxon sum-of-rank test. For categorical variables, the current study used frequency (proportion) to report their distributions in the two CAC groups while Chi-square tests were employed to test the significances of inter-group differences. Binary logistic regression was employed to analyze the unadjusted association of smoking history, current smoking, alcohol consumption history and current alcohol consumption with the presence and severe CAC as well as the associations of aforementioned smoking and alcohol consumption-related variables with CAC adjusting for confounders. Interaction term between alcohol consumption history and current smoking status was added to analyze the interaction effect of alcohol consumption on the association of smoking and CAC.

This study partitioned patients into three groups according to their CACS (CACS = 0, 0 < CACS < 1000, CACS ≥1000). Cumulative odds logistic regression was used for analyzing the association of the association of smoking-related histories like smoking history and current smoking with severe CAC as well as the association of alcohol consumption-related histories like alcohol consumption history and current alcohol consumption. Interaction terms of alcohol consumption history and current smoking status were added in the model to analyze the interaction effect of alcohol consumption. Based upon the results above, patients were rearranged into three groups (CACS = 0, 0 < CACS < 1100, CACS ≥1100). Cumulative odds logistic regression was employed to analyze the unadjusted association of smoking history, current smoking status, alcohol consumption history and current alcohol consumption with the presence of and severe CAC as well as the associations of the aforementioned smoking and alcohol consumption-related variables with CAC adjusting for confounders. Interaction terms between alcohol consumption history and current smoking status were again used to analyze the interaction effect of alcohol consumption.

Multiple imputation was employed to tackle missing data. In the imputation stage, fully conditional specification (FCS) logistic regression was used for imputation of discrete variables while FCS predictive mean matching (PMM) was used for imputation of continuous ones. Following statistical analyses on each of the imputed datasets separately, the results generated were pooled via Rubin’s rule if the variable to be pooled followed a *t *distribution or was demonstrated to have asymptotic normality. If the variable to be pooled followed a Chi-square distribution, the D2 method [25] was employed to pool the results.

Statistical Analysis System (SAS) version 9.4 TS1M5 (SAS Institute Inc., Cary, NC, USA) was used for statistical analyses. *p* values < 0.05 are considered statistically significant.

## 3. Results

### 3.1 Characteristics of the Studied Population

Data of 1528 patients were eventually used for analyses. Table 1 summarizes clinical characteristics of the two CAC groups as well as the statistical test results of inter-group differences. Results indicate that compared to those without CAC, patients with CAC had older age, higher levels of free fatty acids, fasting glucose and glycohemoglobin, declined renal function (measured as lower estimated glomerular filtration rate) and lower apoA1 levels. In addition, the proportion of male, hypertensive, diabetic, former or current smokers, former or current alcohol consumers, aspirin and statin users were higher. No significant differences were found in terms of the triglyceride level, height and weight of the two groups. A decreasing trend was observed in low-density lipoprotein cholesterol (LDL-C) level of CAC patients as compared with those without CAC (*p* = 0.0572). This phenomenon might be associated with the higher proportion of statin users in patients with CAC.

**Table 1.** **Clinical characteristics of studied population**.

Clinical characteristics	CACS = 0 (*n* = 681)	CACS >0 (*n* = 847)	*p* value
Age (Years)	51.02 ± 10.06	59.67 ± 11.25	<0.001
Male gender (%)	455 (66.81%)	654 (77.21%)	<0.001
Hypertension (%)	411 (60.37%)	622 (73.38%)	<0.001
Diabetes (%)	127 (18.66%)	307 (36.25%)	<0.001
Smoking history (%)	273 (40.05%)	424 (50.11%)	<0.001
Current smoking (%)	183 (26.85%)	263 (31.01%)	0.125
Alcohol consumption history (%)	195 (28.67%)	332 (39.21%)	<0.001
Current alcohol consumption (%)	159 (23.33%)	246 (29.03%)	0.026
Aspirin (%)	117 (17.20%)	263 (31.03%)	<0.001
Statins (%)	129 (19.01%)	325 (38.32%)	<0.001
eGFR (mL/(min × 1.73 m^2^))	95.31 ± 15.95	89.51 ± 16.66	<0.001
apoA1 (g/L)	1.43 ± 0.29	1.39 ± 0.30	0.011
Free fatty acid (mmol/L)	0.48 ± 0.22	0.52 ± 0.23	<0.001
TC (mmol/L)	4.72 ± 1.09	4.53 ± 1.20	<0.001
LDL-C (mmol/L)	2.79 ± 0.84	2.70 ± 1.00	0.057
HDL-C (mmol/L)	1.26 ± 0.37	1.22 ± 0.34	0.062
Height (cm)	169.58 ± 8.70	170.03 ± 7.76	0.298
Weight (kg)	75.62 ± 14.33	75.64 ± 12.92	0.971
Glycohemoglobin (%)	5.66 (5.40, 6.00)	5.90 (5.60, 6.60)	<0.001
TG (mmol/L)	1.48 (1.04, 2.23)	1.44 (1.03, 2.18)	0.327
Blood glucose (mmol/L)	5.28 (4.85, 5.90)	5.63 (5.08, 6.81)	<0.001

Continuous variables that followed normal distributions were reported as mean ± standard deviation and were tested for inter-group differences via *t* tests whereas those that did not follow normal distributions were reported as median (1st quartile, 3rd quartile) and were tested for inter-group differences via Wilcoxon sum-of-rank test. For categorical variables, frequency (proportion) were used for reporting while Chi-square tests were employed to test the significances of inter-group differences. To tackle missing data, the results generated on each imputed dataset were pooled via Rubin’s rule if the variable to be pooled followed a *t* distribution or was demonstrated to have asymptotic normality. If the variable to be pooled followed a Chi-square distribution, the D2 method was employed to pool the results. Abbreviations: apoA1, apolipoprotein A1; eGFR, estimated glomerular filtration rate; HDL-C, high-density lipoprotein cholesterol; LDL-C, low-density lipoprotein cholesterol; TC, total cholesterol; TG, triglyceride; CACS, coronary artery calcification score. “Aspirin” and “Statins” refer to usage of the medications.

### 3.2 Univariable Logistic Regression Analyses of Smoking-Related Histories and Presence of CAC

Table 2 summarizes the unadjusted associations of smoking-related histories and presence of CAC. Results in Table 2 are consistent with those in Table 1, indicating that smoking history was a significant risk factor of presence of CAC, yet the association between current smoking and presence of CAC was insignificant.

**Table 2.** **Results of univariable logistic regression analyses of smoking and presence of CAC**.

Variable	CACS = 0 (*n* = 681)	CACS >0 (*n* = 847)	Regression coefficient	OR (95% CI)	*p* value
Smoking history	273 (40.05%)	424 (50.11%)	0.41	1.50 (1.22, 1.85)	<0.001
Current smoking	183 (26.85%)	263 (31.01%)	0.20	1.23 (0.97, 1.55)	0.086

Abbreviations: CAC, coronary artery calcification; CI, confidence interval; OR, odds ratio; CACS, coronary artery calcification score.

### 3.3 Univariable Logistic Regression Analyses of Smoking-Related Histories and Severe CAC

Table 3 summarizes unadjusted associations of smoking-related histories and severe CAC given the definition of severe CAC as CACS ≥1000. Results indicate that smoking history was significantly associated with severe CAC whereas current smoking was insignificantly associated with severe CAC.

**Table 3.** Results of univariable logistic regression analyses of smoking and severe CAC*.

Variable	CACS <1000 (*n* = 1459)	CACS ≥1000 (*n* = 69)	Regression coefficient	OR (95% CI)	*p* value
Smoking history	663 (45.47%)	34 (48.80%)	0.38	1.46 (1.20, 1.79)	0.000
Current smoking	429 (29.41%)	17 (23.93%)	0.17	1.17 (0.94, 1.46)	0.165

Abbreviations: CAC, coronary artery calcification; CI, confidence interval; OR, odds ratio; CACS, coronary artery calcification score. *Defined as CACS ≥1000.

### 3.4 Univariable Logistic Regression Analyses of Alcohol Consumption-Related Histories and Presence of CAC

Table 4 summarizes unadjusted associations of alcohol consumption-related histories and presence of CAC. Results identified both alcohol consumption history and current alcohol consumption as significant risk factors for the presence of CAC.

**Table 4.** **Results of univariable logistic regression analyses of alcohol consumption and presence of CAC**.

Variable	CACS = 0 (*n* = 681)	CACS >0 (*n* = 847)	Regression coefficient	OR (95% CI)	*p* value
Alcohol consumption history	195 (28.67%)	332 (39.21%)	0.47	1.60 (1.27, 2.02)	<0.001
Current alcohol consumption	159 (23.33%)	246 (29.03%)	0.30	1.34 (1.06, 1.71)	0.015

Abbreviations: CAC, coronary artery calcification; CI, confidence interval; OR, odds ratio; CACS, coronary artery calcification score.

### 3.5 Univariable Logistic Regression Analyses of Alcohol-Related Histories and Severe CAC

Detailed in Table 5, results of this section revealed that both alcohol consumption history and current alcohol consumption were significantly associated with severe CAC.

**Table 5.** Results of univariable logistic regression analyses of alcohol consumption and severe CAC*.

Variable	CACS <1000 (*n* = 1459)	CACS ≥1000 (*n* = 69)	Regression coefficient	OR (95% CI)	*p* value
Alcohol consumption history	500 (34.24%)	28 (40.42%)	0.45	1.57 (1.25, 1.96)	<0.0001
Current alcohol consumption	387 (26.53%)	18 (25.67%)	0.26	1.30 (1.03, 1.64)	0.027

Abbreviations: CAC, coronary artery calcification; CI, confidence interval; OR, odds ratio; CACS, coronary artery calcification score. *Defined as CACS ≥1000.

### 3.6 Interaction Effect of Alcohol Consumption History on the Association of Current Smoking and Presence of CAC

Table 6 summarizes the association of alcohol consumption history as well as current smoking with the presence of CAC when factoring in the interaction of alcohol consumption history and current smoking. Results indicated that after adjusting for confounders, both alcohol consumption history and current smoking were associated with the presence of CAC. Moreover, the association of current smoking and presence of CAC was influenced by alcohol consumption history. To further facilitate the elaboration of the interaction effect, patients with no alcohol consumption history who were not currently smoking were used as reference while current smokers were grouped into two subpopulations: those with and those without alcohol consumption history. Relevant results are summarized in Table 7. Results indicated that while current smokers with or without alcohol consumption history were both exposed to an increased risk of CAC, current smokers with concurrent alcohol consumption history were associated with an increased risk of CAC.

**Table 6.** **Multivariable logistic regression analyses of alcohol consumption history and current smoking with presence of CAC**.

Variable	Regression coefficient	*p* value
Alcohol consumption history*	0.78	<0.001
Current smoking**	0.50	0.016
Alcohol consumption history×Current smoking***	–0.63	0.032
Male gender	0.85	<0.001
Diabetes	0.57	<0.001
Age	0.09	<0.001
LDL-C	0.29	<0.001
Statins	0.84	<0.001

Abbreviations: CAC, coronary artery calcification; LDL-C, low-density lipoprotein cholesterol. “Statins” refer to usage of the medication. *Adjusting for current smoking and its interaction with alcohol consumption history, gender, age, diabetes, LDL-C and use of statins. **Adjusting for alcohol consumption history and its interaction with current smoking, gender, age, diabetes, LDL-C and use of statins. ***Refers to the interaction term of alcohol consumption history and current smoking.

**Table 7.** **Risk of presence of CAC for different subpopulations (non-smokers with no alcohol consumption history as reference)**.

Subpopulation	OR (95% CI)	*p* value*
Smoking (-), Alcohol (-)	Reference	-
Smoking (+), Alcohol (-)	1.66 (1.10, 2.49)	0.016
Smoking (+), Alcohol (+)	1.92 (1.32, 2.81)	<0.001

Abbreviations: CAC, coronary artery calcification; CI, confidence interval; OR, odds ratio. Smoking (-), Alcohol (-): Non-alcohol-consumers who currently do not smoke. Smoking (+), Alcohol (-): Current smokers with no alcohol consumption history. Smoking (+), Alcohol (+): Alcohol consumers who currently smoke. *Adjusting for age, gender, diabetes, low-density lipoprotein cholesterol and use of statins.

### 3.7 Interaction Effect of Alcohol Consumption History on the Association of Current Smoking and Severe CAC

Association of alcohol consumption history and current smoking with severe CAC when interaction was factored in are summarized in Table 8. Results indicated that both alcohol consumption history and current smoking were risk factors for severe CAC. In addition, *p* value of the interaction term was slightly larger than 0.05, suggesting the probable presence of an interaction effect of alcohol consumption history on the association of current smoking with severe CAC when severe CAC was defined as CACS ≥1000. In other words, similar to the case in the presence of CAC, association of current smoking with severe CAC might also be influenced by alcohol consumption history. For clarity, patients who did not smoke currently and had no alcohol consumption history were used as reference and those either with or without alcohol consumption history were grouped into the two foregoing subpopulations. Odds ratios of the subpopulations for severe CAC are detailed in Table 9. Similar to the situation in the presence of CAC, results of this section indicated while current smokers with or without alcohol consumption history were both exposed to an increased risk of severe CAC, current smokers with concurrent alcohol consumption history were associated with an increased risk of severe CAC.

**Table 8.** Multivariable logistic regression analyses of alcohol consumption history and current smoking with severe CAC*.

Variable	Regression coefficient	*p* value
Alcohol consumption history**	0.70	<0.001
Current smoking***	0.39	0.046
Alcohol consumption history×Current smoking****	–0.51	0.059
Male gender	0.93	<0.001
Diabetes	0.62	<0.001
Age	0.09	<0.001
LDL-C	0.31	<0.001
Statins	0.77	<0.001

Abbreviations: CAC, coronary artery calcification; LDL-C, low-density lipoprotein cholesterol. “Statins” refer to usage of the medication. *Defined as CACS ≥1000. **Adjusting for current smoking and its interaction with alcohol consumption history, gender, age, diabetes, LDL-C and use of statins. ***Adjusting for alcohol consumption history and its interaction with current smoking, gender, age, diabetes, LDL-C and use of statins. ****Refers to the interaction term of alcohol consumption history and current smoking.

**Table 9.** Risk of severe CAC* for different subpopulations (non-smokers with no alcohol consumption history as reference).

Subpopulation	OR (95% CI)	*p* value**
Smoking (-), Alcohol (-)	Reference	-
Smoking (+), Alcohol (-)	1.47 (1.01, 2.15)	0.046
Smoking (+), Alcohol (+)	1.78 (1.25, 2.53)	<0.001

Abbreviations: CAC, coronary artery calcification; CI, confidence interval; OR, odds ratio. Smoking (-), Alcohol (-): Non-alcohol-consumers who currently do not smoke. Smoking (+), Alcohol (-): Current smokers with no alcohol consumption history. Smoking (+), Alcohol (+): Alcohol consumers who currently smoke. *Defined as CACS ≥1000. **Adjusting for age, gender, diabetes, low-density lipoprotein cholesterol and use of statins.

### 3.8 Univariable Logistic Regression Analyses of Smoking-Related Histories and Severe CAC after Regrouping

In order to further analyze the association of smoking, alcohol consumption and severe CAC while investigating the interaction effect of alcohol consumption history, a regrouping of patients was conducted, i.e., grouping the patients into three groups according to CACS (CACS = 0, 0 < CACS < 1100, CACS ≥1100), which served as the basis of the following analyses. Table 10 summarizes results of univariable analyses of associations of smoking-related histories and severe CAC. Results again revealed that smoking history was a risk factor of severe CAC whereas current smoking was not significantly associated with severe CAC.

**Table 10.** Results of univariable logistic regression analyses of smoking-related histories and severe CAC*.

Variable	CACS <1100 (n = 1471)	CACS ≥1100 (n = 57)	Regression coefficient	OR (95% CI)	*p* value
Smoking history	668 (45.44%)	29 (50.30%)	0.39	1.48 (1.21, 1.81)	<0.001
Current smoking	433 (29.41%)	13 (22.68%)	0.16	1.17 (0.94, 1.47)	0.162

Abbreviations: CAC, coronary artery calcification; CI, confidence interval; OR, odds ratio; CACS, coronary artery calcification score. *Defined as CACS ≥1100.

### 3.9 Univariable Logistic Regression Analyses of Alcohol Consumption-Related Histories and Severe CAC after Regrouping

Results of univariable analyses of associations of alcohol consumption-related histories and severe CAC are summarized in Table 11. They also revealed that both alcohol consumption history and current alcohol consumption were significantly associated with severe CAC.

**Table 11.** Results of univariable logistic regression analyses of alcohol consumption-related histories and severe CAC*.

Variable	CACS <1100 (*n* = 1471)	CACS ≥1100 (*n* = 57)	Regression coefficient	OR (95% CI)	*p* value
Alcohol consumption history	505 (34.34%)	22 (39.00%)	0.44	1.56 (1.25, 2.00)	<0.001
Current alcohol consumption	391 (26.55%)	14 (25.02%)	0.26	1.30 (1.03, 1.64)	0.026

Abbreviations: CAC, coronary artery calcification; CI, confidence interval; OR, odds ratio; CACS, coronary artery calcification score. *Defined as CACS ≥1100.

### 3.10 Interaction Effect of Alcohol Consumption History on the Association of Current Smoking and Severe CAC after Regrouping

Table 12 summarizes the results of the adjusted association of alcohol consumption history and current smoking with severe CAC as well as the interaction effect after regrouping. Results indicate that both alcohol consumption history and current smoking status were significantly associated with severe CAC after adjusting for confounders. A significant interaction effect of alcohol consumption history was observed after regrouping, confirming its presence. Similar to the analyses performed above, this study also chose current non-smokers free of alcohol consumption history as reference and calculated the risks of severe CAC for current smokers with alcohol consumption history and current smokers without alcohol consumption history, which are summarized in Table 13. Results indicate that after adjusting for confounders, current smoking was a significant risk factor in both alcohol consumers and non-alcohol consumers.

**Table 12.** Multivariable logistic regression analyses of alcohol consumption history and current smoking with severe CAC* after regrouping.

Variable	Regression coefficient	*p* value
Alcohol consumption history**	0.72	<0.001
Current smoking***	0.42	0.031
Alcohol consumption history×Current smoking****	–0.55	0.046
Male gender	0.89	<0.001
Diabetes	0.59	<0.001
Age	0.09	<0.001
LDL-C	0.30	<0.001
Statins	0.78	<0.001

Abbreviations: CAC, coronary artery calcification; LDL-C, low-density lipoprotein cholesterol. “Statins” refer to usage of the medication. *Defined as CACS ≥1100. **Adjusting for current smoking and its interaction with alcohol consumption history, gender, age, diabetes, LDL-C and use of statins. ***Adjusting for alcohol consumption history and its interaction with current smoking, gender, age, diabetes, LDL-C and use of statins. ****Refers to the interaction term of alcohol consumption history and current smoking.

**Table 13.** Risk of severe CAC* for different subpopulations after regrouping (non-smokers with no alcohol consumption history as reference).

Subpopulation	OR (95% CI)	*p* value**
Smoking (-), Alcohol (-)	Reference	-
Smoking (+), Alcohol (-)	1.52 (1.04, 2.23)	0.031
Smoking (+), Alcohol (+)	1.81 (1.27, 2.58)	<0.001

Abbreviations: CAC, coronary artery calcification; CI, confidence interval; OR, odds ratio. Smoking (-), Alcohol (-): Non-alcohol-consumers who currently do not smoke. Smoking (+), Alcohol (-): Current smokers with no alcohol consumption history. Smoking (+), Alcohol (+): Alcohol consumers who currently smoke. *Defined as CACS ≥1100. **Adjusting for age, gender, diabetes, low-density lipoprotein cholesterol and use of statins.

## 4. Discussion

CAC refers to calcium deposition on the coronary arterial wall and is an important type of vascular calcification. Vascular calcification can be classified into (atherosclerotic) intimal calcification, medial calcification and genetic calcification [26]. Being a measure quantifying the severity of CAC, CACS can also be used for measuring coronary atherosclerotic burden [27] and overall atherosclerotic burden [28].

Generally, studies to date investigating the association of smoking and CAC have confirmed their associations in multiple respects, despite the attenuation or even lack of presence of such associations in certain populations. Molina* et al*. [15] conducted a retrospective study on 1162 South Asian individuals in North America. Results pointed out that smoking history (current or former smoking) was a risk factor for CAC. Wetscherek *et al*. [29] also found smoking history to be a risk factor of CAC in univariate analysis. Moreover, Zhang *et al*. [30] concluded that smoking history was a risk factor of CAC after adjusting for confounders in a cohort of 4989 asymptomatic individuals who participated in screening for lung cancer at a Chinese cancer hospital. This association was also presented in other cohorts. Prior to Zhang *et al*. [30], Trab *et al*. [31] analyzed data of 163 schizophrenics in Denmark and also found smoking history (defined as current or former smoking) a risk factor of presence of CAC after adjusting for confounders. However, Kiss *et al*. [14] analyzed data of 511 participants of Budakalász Health Survey, a cardiovascular screening program in a central Hungarian town and found that smoking was not significantly associated with CAC after the adjustment of confounders. Yet the concept of “smoking” was not clearly defined in the study by Kiss *et al*. [14]. In all, it can be concluded from prior studies that smoking history is associated with CAC. Our results are consistent with them.

The association of current smoking status and CAC has also been examined. The Jackson Heart Study conducted by Oshunbade *et al*. [32] enrolled 4432 black adults free of coronary heart disease and recorded their baseline smoking status as well as their CACS at follow-up. Results indicated that compared with non-smokers, former smokers or current smokers at baseline were exposed to a significantly increased risk of CAC at follow-up. However, the association of current smoking and presence of CAC at the same time point was not investigated in the Jackson Heart Study. Lee *et al*. [33] found on a cohort of 1914 Korean patients with CKD that compared with never smokers, current smokers were associated with a significantly increased risk of CAC after adjusting for confounders. Multivariable analysis results from this study are different from those of the aforementioned studies, especially that of Lee *et al*. [33] This is exemplified in several aspects. (1) Study population of our study differs from the study by Lee *et al*. [33]. While both the current study and the study by Lee *et al*. [33] are conducted on eastern Asian populations, this study is conducted on patients visiting a hospital primarily for cardiovascular diseases while the population studied by Lee *et al*. [33] was confined to patients with CKD. (2) Comparisons of the two studies were different. This study compared the risk of presence of CAC of patients who smoked currently against those who did not smoke currently while the comparison conducted by Lee *et al*. [33] was current smokers vs. never smokers. (3) Last but not least, the confounders which were adjusted for were different. While Lee *et al*. [33] did not adjust their results for any variable related to alcohol consumption, alcohol consumption history was adjusted in this study.

Researchers have also conducted investigations of the association between smoking and severe CAC. Capisizu *et al*. [12] conducted a single-center study on 222 individuals. Results indicated smoking history was significantly associated with severe CAC (defined as CACS ≥400). The aforementioned study conducted by Kiss *et al*. [14] on 511 Budakalász Health Survey participants also found smoking significantly associated with CACS ≥300 after adjusting for confounders. But, as has already been pointed out in this article, the concept of “smoking” was not clearly defined [14]. In all, with a much larger sample size than previously conducted studies, this study also found that smoking history was significantly associated with severe CAC, despite different definitions of the disease.

Research on current smoking status and severe CAC is also present. Similar to Kiss *et al*. [14], Bagyura *et al*. [13] also investigated data of Budakalász Health Survey and eventually enrolled 280 participants with age ≥35 years old (for males) or ≥40 years old (for females) and neither history of cardiovascular events or diseases (including chronic heart failure, angina pectoris, myocardial infarction, PCI, CABG, cardiomyopathy, peripheral vascular diseases and arrythmias) nor inflammatory disorders (defined as use of glucocorticoids and other immunomodulators as well as C-reactive protein >30 mg/L). Univariable analyses revealed that current smoking (defined as at least smoking one cigarette per day at the time of the study) was not associated with severe CAC (defined as CACS >100). However, results of multivariable analyses found that despite this insignificance lingered after adjusting for age, gender, interaction of neutrophil/lymphocyte ratio and tertile of visceral adiposity index as well as body mass index, further adjustment of either cardiometabolic diseases (hyperlipidemia, hypertension and diabetes) or cardiometabolic diseases + glycohemoglobin + C-reactive protein attained results indicating that current smoking was significantly associated with severe CAC, with OR stood at 3.20 and 3.97 respectively [13]. Differences exist between results of the study by Bagyura *et al*. [13] and the current study, which may be associated with the following factors. (1) Sample size of this study outnumbers that of by Bagyura *et al*. [13]. (2) Studied population of this study was different from that by Bagyura *et al*. [13] in the following aspects. (A) While this study was conducted on Chinese individuals, Bagyura *et al*. [13] analyzed data from an eastern European population. (B) Studied participants of Bagyura *et al*. [13] came from a geographically delimited region (a town) while participants of our study came from all over China. (C) Bagyura *et al*. [13] studied populations from a health survey while this study was based on patients visiting a hospital with special expertise on Cardiology. (3) Exclusion criteria of the study by Bagyura *et al*. [13] confined it to populations free of cardiovascular diseases or events while this study only excluded patients with prior PCI, CABG because of the inability to calculate CACS of PCI recipients or the altered coronary flow that may exert influence on CAC in patients with CABG history. (4) Differences in the definition of severe CAC existed between the two studies. (5) Alcohol consumption history was not adjusted for with respect to the result obtained by Bagyura *et al*. [13] whereas our study adjusted the results for it. Therefore, these comparisons indicate that race and comorbidities may influence the association of current smoking and severe CAC, a condition with possibly different definitions among studies. The types of confounders adjusted for also has an impact on the results.

The association of alcohol consumption and CAC is more obscure than that of smoking and CAC. Studies investigating the former have generally revealed the presence of association. McClelland *et al*. [10] investigated 6814 American individuals aged between 45 and 84 years old and were free from clinically apparent cardiovascular diseases, including White, African American, Hispanics and Chinese American. Results indicated that after adjusting for confounders, amount of liquor consumption correlated significantly with CAC. However, for usual alcohol consumption, the amount of alcohol consumed and former alcohol consumption were both insignificantly associated with CAC after adjustment of confounders. When it comes to the specific type of alcohol consumed, different degrees of amount of wine, beer, liquor consumed as well as total amount of alcohol consumed were not significantly associated (including associated in a J-shaped manner) with CAC after adjusting for confounders. Compared with those that never consumed liquor, populations that consumed liquor in the month prior to the study took place suffered from a significant increase of risk of the presence of CAC. Moreover, the association of liquor consumption and presence of CAC was generally found to be present among Chinese individuals [10]. The CARDIA study enrolled healthy black and white individuals aged between 33 and 45 years old [19]. Results indicated that heavy alcohol drinking (≥14 drinks per week) was independently associated with CAC. A statistically significant trend regarding the amount of alcohol consumed and likelihood of CAC in the entire population was observed. This trend was also statistically significant in both black male and black female subpopulations while the significance was absent in white male and white females. Liquor consumption also significantly correlated with the presence of CAC [19]. However, in a cohort of 731 healthy American military personnels that aged between 39 to 45 years old and were almost exclusively white (72%), researchers found none of several consumption-related variables (alcohol consumption, type or alcohol consumed and amount of alcohol consumed) were significantly associated with the presence of CAC [22]. The population studied by McClelland *et al*. [10] shared a fraction of similarity with the current study in that Chinese Americans in the study by McClelland *et al*. [10] accounted for 11.81% of the entire study population while the current study was solely based on Chinese individuals. This can explain the difference obtained between the study by McClelland *et al*. [10] and this study. This study is a retrospective study that utilized data from medical records where type of alcohol consumed was not recorded. However, the study found alcohol consumption history significantly associated with presence of CAC after adjusting for confounders. The discrepancy between results obtained by this study and the study by McClelland *et al*. [10] may be associated with racial difference between the study populations. More specifically, McClelland *et al*. [10] found liquor consumption to be associated with CAC in the Chinese population. This might have contributed to the significance found in this study.

The current study also investigated the association of alcohol consumption and the severity of CAC, which was also studied by prior studies. For instance, McClelland *et al*. [10] found consumption of more than two drinks of liquor per day was associated with the severity of CAC in individuals with CACS >0; consumption of more than two drinks of liquor per day was also associated with the drastic change in CAC during follow-up (>30 units per year) in individuals with baseline CACS >0 [10]. However, there are also studies that failed to find associations between alcohol consumption and severe CAC. A study has found a lack of association between alcohol consumption and amount of CAC in a cohort of asymptomatic individuals at high risk of coronary heart disease [21]. Vliegenthart *et al*. [23] found that the amount of alcohol consumed to be U-shaped correlated with severe CAC, despite the statistical insignificance of the correlation. They analyzed data of 1795 participants from the Rotterdam Coronary Calcification Study and found that compared with non-drinkers, participants who drank ≤one drink per day had lower odds of being afflicted by severe CAC (defined as CACS >400) (OR = 0.60), while odds ratios for those who drank 1–2 drinks per day and >2 drinks per day stood at 0.51 and 0.90 respectively. However, none of the trio were statistically significant. While both the current study and the study by Vliegenthart *et al*. [23] defined severe CAC as a situation where CACS exceeded a certain threshold, differences in the threshold existed. This, along with the difference in study population (i.e., the one by Vliegenthart *et al*. [23] was residents in Rotterdam, The Netherlands while the one of this study was Chinese), may have contributed to the differences in the current study and the one by Vliegenthart *et al*. [23].

Until now, the majority of studies investigating the association of smoking and alcohol consumption with CAC analyzed them independently. However, evidence from basic biological studies have casted light on the potential dependence of the association of smoking and vascular calcification on drinks containing alcohol. The aforementioned animal study by Liou *et al*. [24] found that xanthohumol, a chemical found in hops, an indispensable ingredient in producing beer, could alleviate vascular calcification mediated by vitamin D and nicotine. Therefore, beer consumption could influence the association between smoking and vascular calcification. It is therefore worthwhile to investigate in human subjects if alcohol consumption could influence the association between smoking and CAC. In other words, clinical evidence regarding the potential interaction effect of alcohol consumption on the association of smoking and CAC deserves to be gathered, which was part of the current study. Lee *et al*. [33] found that compared with never smokers, current smokers were exposed to a significant increase in risk of CAC while the risk increment of former smokers was insignificant. This study therefore analyzed if the interaction effect of alcohol consumption history existed with respect to the association of current smoking and CAC. While reaffirming that both current smoking and alcohol consumption history were risk factors of the presence of and severe CAC, results of this study found current smokers with concomitant alcohol consumption history were afflicted by an increase in risk of both presence of and severe CAC. These results also suggest that detailed biological mechanisms of the effect of alcohol (including ethanol and other chemicals that coexist) and smoking on vascular calcification should be further investigated. From a public health point of view, while significant weights have been laid upon tobacco control when it comes to the prevention of CAC and coronary atherosclerosis, results of this study have suggested a non-negligible weight of alcohol consumption in the formulation of policies tackling atherosclerotic diseases. When it comes to the prevention of CAC, emphasis should be laid upon current smokers who simultaneously had alcohol consumption history. From a clinical point of view, results of this study call on clinicians to be aware of the increased risk of CAC associated with both smoking and alcohol consumption, especially among those who simultaneously have a history of alcohol consumption and currently smoke.

Limitations of this study include: (1) This study is a single-center study based on patients that visited a hospital with expertise on Cardiology. Whether the results generalize to other populations remains to be investigated. (2) This study is a retrospective study based on medical records with the possibility of recall bias. In addition, the amount of information available has provided room for unmeasured confounders. (3) As a measure of CAC, CACS itself is subject to limitations. For instance, patients having undergone PCI (especially coronary stent implantation) are ineligible for calculating CACS.

Our study has provided clinical evidence of the interaction effect of alcohol consumption on the association between current smoking and CAC. Future research could be oriented in the following directions: (1) Further explore the underlying biological mechanism of the interaction effect. (2) Further examine the association of smoking and alcohol consumption with the onset and exacerbation in cohorts who have their CAC repeatedly gauged. (3) Examine the necessity to provide targeted care by setting more stringent goals in controlling atherosclerosis for current smokers with alcohol consumption history.

## 5. Conclusions

Both smoking history and alcohol consumption history correlated positively with the presence of and severe CAC. Alcohol consumption history has been shown to alter the association of current smoking and both the presence of and severe CAC. Alcohol consumption history was associated with a more pronounced risk for the presence of and severe CAC in current smokers.

## Availability of Data and Materials

Data of the current study could be made available in response to reasonable request proposed to the corresponding author.
